# Occult Hepatitis B Virus infection in ART-Naive HIV-Infected Patients seen at a Tertiary Care Centre in North India

**DOI:** 10.1186/1471-2334-10-53

**Published:** 2010-03-07

**Authors:** Swati Gupta, Sarman Singh

**Affiliations:** 1 Division of Clinical Microbiology, Department of Laboratory Medicine, All India Institute of Medical Sciences, New Delhi-110029, India

## Abstract

**Background:**

Co-infections of hepatitis B and C viruses are frequent with HIV due to shared routes of transmission. In most of the tertiary care health settings, HIV reactive patients are routinely tested for HBsAg and anti-HCV antibodies to rule out these co-infections. However, using the routine serological markers one can only detect active HBV infection while the occult HBV infection may be missed. There is insufficient data from India on HIV-HBV co-infection and even scarce on occult HBV infection in this group.

**Methods:**

We estimated the burden of HBV infection in patients who were tested positive for HIV at a tertiary care centre in north India. We also attempted to determine the prevalence and clinical characteristics of occult HBV infection among these treatment-naïve patients and compare their demographic features with other HIV patients. During a period of 6 years between January 2002 to December 2007, 837 HIV positive patients (631 males and 206 females (M: F :: 3.06:1) were tested for serological markers of HBV (HBsAg) and HCV (anti-HCV antibodies) infections in our laboratory. For comparison 1000 apparently healthy, HIV-negative organ donors were also included in the study. Data on demographics, sexual behaviour, medical history, laboratory tests including the serum ALT and CD4 count of these patients were recorded. A sub-group of 53 HBsAg negative samples from HIV positive patients were assessed for anti-HBs, anti-HBc total (IgG+IgM) and HBV-DNA using a highly sensitive qualitative PCR and analysed retrospectively.

**Results:**

Overall, 7.28% of HIV positive patients showed presence of HBsAg as compared to 1.4% in the HIV negative control group. The prevalence of HBsAg was higher (8.55%) in males than females (3.39%). The study revealed that occult HBV infection with detectable HBV-DNA was prevalent in 24.5% of patients positive for anti-HBc antibodies; being 45.5% in HBsAg negative patients. Most importantly the occult infection was seen in 20.7% patients who were positive for anti-HBs antibodies. However, in none of the seronegative patient HBV-DNA was detected. Five of the nine HBV-DNA positive (55.6%) patients showed raised alanine aminotransferase levels and 66.7% had CD4^+ ^T cell counts below 200 cells/cumm.

**Conclusions:**

High prevalence of HIV-HBV co-infection was found in our patients. A sizeable number of co-infected patients remain undiagnosed, if only conventional serological markers are used. Presence of anti-HBs antibodies was not a reliable surrogate marker to rule out occult HBV infection. The most reliable method to diagnose occult HBV co-infection in HIV seropositive patients is the detection of HBV-DNA.

## Background

Human immunodeficiency virus (HIV) establishes a chronic and latent infection in the body that induces extensive damage to the immune system through virus-related as well as indirect pathogenic mechanisms [1]. HIV infected individuals show not only a quantitative depletion of CD4+ T cells but also an overall immune dysregulation. Hepatitis B virus (HBV) is a frequent co-contaminant with HIV, mainly because both share common modes of transmission. Whether the presence of one facilitates the sexual transmission of the other is a matter under investigation, but both are easily transmitted through infected blood, unsafe injections and equipments. Over the years, much evidence has accumulated that co-infection with HIV significantly modifies the natural history of HBV infection [2,3]. Highly active antiretroviral therapy (HAART) is well known to prolong the survival of HIV-infected individuals which allows a longer time for cirrhosis to develop in HBV co-infected patients. Therefore, HAART increases the relative proportion of deaths attributable to liver disease in these patients. HAART may also have a major impact on HBV co-infection because of restoration of specific and non-specific immune responses and decrease of aberrant activation and dysregulation of the immune system [4]. Withdrawal or development of resistance to drugs that are dually active against both HIV and HBV has been associated with reactivation of HBV infection and with flares of liver enzyme elevations and hepatic decompensation in patients with advanced liver disease [5,6].

At our institute all HIV reactive patients are also screened for hepatitis B surface antigen (HBsAg), if treating physician has any suspicion of co-infection. However, not all hepatitis B virus co-infections are symptomatic and even routine serological markers can miss the diagnosis of HBV disease. Those patients who turn out to be positive for HBV-DNA in the absence of serum HBsAg are known as occult hepatitis B virus (HBV) infections [7]. Occult HBV infection is characterised by long lasting persistence of HBV-DNA in serum, and/or hepatic tissues of individuals negative for serum HBsAg. The presence of antibodies to HBV core antigen (anti-HBc-IgM) is now recognized as a better indicator of progressing occult HBV infection. But, recent estimates suggest that up to 20% of individuals with occult HBV could be negative even for anti-HBc antibodies or any other serological indicator of exposure to HBV [7]. Some studies also show that the detection of naturally acquired antibodies to HBsAg (anti-HBs) does not exclude the existence of occult infection [8], indicating that diagnosis of occult HBV co-infection is a challenging task for laboratory physicians.

True prevalence and clinical significance of occult HBV infections is scarcely reported in the literature. Occult HBV has been implicated in the transmission of the HBV via transfusion of blood and blood products [9,10] and transplanted solid organs [11] leading to hepatitis in recipients. Reactivation of occult infection has also been reported in hematologic malignancies [12], and immunosuppressed states [13] with reconstitution of immune surveillance associated with activation of virus and cytotoxic T-cell mediated hepatitis. However, the prevalence of occult HBV infection in HIV-infected persons is more so controversial because of the lack of standardization of diagnostic methods and fewer prospective studies. Studies have reported rates of occult HBV markedly varying from as low as nil to as high as 89.5% [14-17] in these patients. However, there are only a few reports of occult HBV infection, as such from India and even scarce in HIV seropositive patients. In this study, we estimated the burden of HBV in patients who tested positive for HIV at a tertiary care centre in north India. We also attempted to determine the prevalence and clinical characteristics of occult HBV among HIV-infected, treatment- naïve patients.

## Methods

### Study population

The study was carried out in the Clinical Microbiology Division, Department of Laboratory Medicine at the All India Institute of Medical Sciences (AIIMS). At this referral institute patients suspected with HIV associated illnesses are seen in the medicine out-patient clinics. Here they are counselled by a social worker and informed consent is taken for testing. As a routine, our laboratory follows the World Health Organization (WHO) testing strategies for HIV test followed by post-test counselling of the patients and this is documented. As an investigative protocol at the institute, all confirmed HIV positive patients are screened for Hepatitis B (HBsAg) and C viruses (anti-HCV antibodies) as a part of their pre-ART work-up beside other tests. A retrospective analysis was performed on data collected from January 2002 to December 2007 in the laboratory.

### Laboratory analysis

Serum samples from 837 proven HIV seropositive patients (WHO testing strategy III, as mentioned above) were collected, labelled with a laboratory identification serial number and stored at -20°C in separate aliquots for further testing upto 10 years or till the storage space is available. From these samples one aliquot was tested for HBsAg and for anti-HCV antibodies using enzyme linked immunosorbent assay kits (*bio Merieux*, France). All serum samples were tested in duplicate.

From the stored serum samples of 776 HIV positive but HBsAg negative patients, we selected 53 serum samples randomly, for reasons of feasibility and availability of reagents and quantity of samples. These were tested for occult HBV infection viz. for anti-HBs antibodies (*bio Merieux*, France), anti HBc total (IgG+ IgM; *biokit*, Spain) and a qualitative PCR for HBV DNA (*SORPOline*™ HBV end-point PCR kit) was also done. This PCR kit has a reported sensitivity of 10-30 copies of HBV. Risk factors and transaminase levels weer ascertained at the time of sample collection. Data on demographics, sexual behaviour, medical history, other laboratory tests including., serum ALT and CD4+ count were also analysed from the records. All tests were performed in accordance with the institutional ethical guidelines at that time. Data of 1000 age and sex matched HIV-negative healthy organ donors was also analysed to compare the prevalence of hepatitis markers. These organ donors are screened with due consent for viral markers as a routine work-up for their family members requiring organ transplantation, as described earlier [18].

### Statistical analysis

Comparison of proportions among different groups and characteristics was done using Fisher's exact test. p < 0.05 was taken as significant.

## Results

A retrospective analysis was performed on laboratory data from 837 HIV sero-reactive patients who were tested in our laboratory from January 2002 to December 2007. Among the study subjects, there were 631 males and 206 females (M: F - 3.06:1). These patients aged between 1-73 years (median 32 years). Overall, HBsAg positivity (HBV co-infection) was seen in 61 (7.28%) HIV positive patients. This rate was highly significant (p < 0.0001) when compared to 1.4% in the control group which comprised of age and sex matched HIV-negative healthy persons. Further, out of the 61 serologically (HBsAg positive) reactive subjects for HIV and HBV co-infection; 54 were males and 7 female. Our patients were in the age range of 14-69 years (median 36 years), but the HBsAg positivity rate was significantly higher in males (8.55%) as compared to 3.39% in female patients (p = 0.0129). Majority (635 of 837) of the HIV positive patients were aged between 21-40 years. This trend was similar in HIV-HBV co-infected patients also (table 1).

**Table 1:** Comparative distribution of HIV positive patients by age and gender divided into two groups on the basis of  presence or absence of HBsAg.

HBs-Ag positive (n = 61)					
	**Age Groups (in years) & no.(%)**				
		
**Sex**	**<20**	**21-30**	**31-40**	**>41**	**Total**

Male	1 (1.6)	17 (27.9)	21 (34.4)	15 (24.6)	54 (88.5)

Female	1 (1.6)	2 (3.3)	2 (3.3)	2 (3.3)	7 (11.5)

Total	2 (3.2)	19 (31.2)	23 (37.7)	17 (27.9)	61 (100)

**HBsAg Negative (n = 776)**					

**Sex**	**Age Groups (in years) & no. (%)**				
		
	**<20**	**21-30**	**31-40**	**>41**	**Total**

Male	29 (3.7)	191 (24.6)	238 (30.7)	119 (15.3)	577 (74.4)

Female	12 (1.5)	97 (12.5)	67 (8.6)	23 (3)	199 (25.6)

Total	41 (5.2)	288 (37.1)	305 (39.3)	142 (18.3)	776 (100)

**Total HIV positive patients (n = 837)**					

**Sex**	**Age Groups (in years) & no. (%)**				
		
	**<20**	**21-30**	**31-40**	**>41**	**Total**

Male	30 (3.5)	208 (24.8)	69 (8.2)	25 (3)	631 (75.4)

Female	13 (1.5)	99 (11.8)	69 (8.2)	25 (3)	206 (24.6)

Total	43 (5.1)	307 (36.7)	328 (39.2)	159 (19)	837 (100)

Note the number in parenthesis is percentage distribution in each group.

Among the 53 HBsAg negative samples tested for occult HBV infection, 41 (77.4%) were males and 12 (22.6%) females. The age group of these patients ranged from 21-59 years (median 32 years). The patients belonged to different risk groups, but the heterosexual risk group was the most predominant. None of these patients gave history of vaccination against HBV. Serological markers of HBV exposure (other than HBsAg, i.e. either anti-HBs or anti-HBc antibody positivity) were seen in 45.3% (24 out of 53) patients. While in 29 patients no marker was detected (table 2). Among these 53 patients, 20.7% (11) showed anti-HBs antibodies and 35.8% (19) anti-HBc (total) antibodies. There was no statistical difference in the prevalence of these markers in two genders; being 46.3% in males and 41.7% in females (table 2). Of the various risk factors for occult HBV infection, male gender combined with low CD4^+ ^counts below 200 cells/cumm and raised alanine aminotransferase levels (>40 IU/L) were most significant (table 3).

**Table 2:** Characteristics and positivity rate of other HBV serological markers in HBsAg-negative HIV-seropositive patients in whom the occult HBV infection rate was investigated.

	*n*	Anti-HBs and/or anti-HBc pos (%)	Anti-HBc pos (%)	Anti-HBs pos (%)	Only Anti-HBc pos (%)	Only Anti-HBs pos (%)	No HBV marker detected (%)
**Overall**	53	24 (45.3)	19 (35.8)	11 (20.7)	13 (24.5)	5 (9.4)	29 (54.7)

**Sex**							

**Male**	41	19 (46.3)	16 (39.0)	8 (19.5)	11 (26.8)	3 (7.3)	22 (53.6)

**Female**	12	5 (41.7)	3 (25)	3 (25)	2 (16.7)	2 (16.7)	7 (58.3)

**Risk factors**^a^							

**Heterosexual**	46	20 (43.5)	16 (34.8)	8 (17.4)	12 (26.1)	4 (8.7)	26 (56.6)

**Transfusion**	3	2 (66.7)	1 (33.3)	2 (66.7)	0	1 (33.3)	1 (33.3)

**IVDU**	2	2 (100)	2 (100)	1 (50)	1 (50)	0	0

**CD4 cell counts**							

**≤ 200**	35	15 (42.8)	12 (34.3)	4 (11.4)	11 (31.4)	3 (8.5)	20 (57.1)

**201-499**	12	7 (58.3)	5 (41.7)	6 (50)	1 (8.3)	2 (16.7)	5 (41.6)

**≥ 500**	6	2 (33.3)	2 (33.3)	1 (16.7)	1 (16.7)	0	4 (66.7)

**ALT levels**							

**Normal**	30	14 (46.6)	11 (36.6)	8 (26.6)	6 (20)	4 (13.3)	16 (53.3)

**Raised^b^**	23	10 (43.4)	8 (34.7)	3 (13.0)	7 (30.4)	1 (4.3)	13 (56.5)

**Other co-infections**							

**HCV pos^c^**	5	4 (80)	3 (60)	2 (40)	2 (40)	1 (20)	1 (20)

**TB pos^d^**	17	6 (35.3)	5 (29.4)	3 (17.6)	3 (17.6)	1 (5.9)	11 (64.7)

Note: n, total no. tested; HCV, hepatitis C virus; TB, Mycobacterium tuberculosis; ALT, alanine aminotransferase; IVDU, injection drug use. Determined by Fisher's exact test for proportions

^a ^In 2 patients no risk factor could be ascertained.

^b ^Defined as serum ALT levels >40 IU/L

^c ^Determined by anti-HCV IgM antibodies

^d ^Diagnosis of tuberculosis was based on clinical suspicion, chest X-ray findings and sputum mycobacteriology

**Table 3:** Characteristics and HBV-DNA positivity in HBsAg negative HIV positive patients with or without the presence of other marker of HBV infection.

	Serological Status		HBV-DNA Status*		
	**Positive for any marker** **n = 24**	**p^a^**	**Pos (%)** **n = 9**	**Neg (%)** **n = 15**	**p^a^**

**Sex**					

Male	19	0.6	7 (77.8)	12 (80)	1.0

Female	5		2 (22.2)	3 (20)	

**Risk factors**					

Heterosexual	20	0.3	8 (88.9)	12 (80)	1.0

Transfusion	2		1 (11.1)	1 (6.7)	

IVDU	2		0	2 (13.3)	

**CD4 cell counts**					

≤ 200	15	0.03	6 (66.7)	9 (60)	1.0

>200	9		3 (33.3)	6 (40)	

**ALT levels**					

Raised^b^	9	0.73	5 (55.6)	7 (46.7)	1.0

**Other infections**					

HCV pos^c^	4	1.0	1 (11.1)	3 (20)	1.0

TB pos^d^	6	1.0	3 (33.3)	3 (20)	0.63

Note: Figures indicate number and percentage (in parenthesis) of patients unless indicated otherwise. n = total no. tested; HCV, hepatitis C virus; TB, tuberculosis; ALT, alanine aminotransferase; IVDU, injection drug use

^a ^Determined by Fisher's exact test for proportions

^b ^Defined as serum ALT levels >40 IU/L

^c ^Determined by anti-HCV IgM antibodies

^d ^Diagnosis of tuberculosis was based on clinical suspicion, chest X-ray findings and sputum mycobacteriology

* In none of the remaining 29 patients, who were negative for all HBV seromarkers, HBV DNA was detected.

HCV co-infection was seen in only 5 (9.4%) of the 53 HBsAg negative patients. This rate was only marginally lower (6.2%) in 61 HBsAg positive pool. Among the HIV-HCV co-infected patients, positivity for anti-HBs/anti-HBc was seen in 80% samples as compared to 41.7% in HCV negative samples (p = 0.163). Tuberculosis (TB) is a common infection in Indian HIV positive patients; hence we compared the presence of these serological markers of occult HBV infection in HIV-TB co-infected and uninfected patients. We found that presence of either of the HBV markers was seen in 35.2% of TB positive patients and in 50% of TB uninfected patients. This difference was statistically insignificant. Anti-HBc alone (in absence of anti-HBs) as a marker of occult infection was seen in 11.3% of samples tested and was associated with lower CD4^+ ^levels below 200 cells/cumm in 84.6% patients (p = 0.032) as compared to higher CD4^+ ^levels (table 3). None of the other characteristics showed a significant association with anti-HBc positivity.

HBV-DNA was positive in 45.3% (24/53) samples which were negative for HBsAg but positive for other HBV serological marker. As mentioned above, none of 29 patients who were negative for all HBV seromarkers was positive for HBV-DNA. In patients who were positive only for anti-HBc antibodies, we found a 24.5% (13/53) prevalence rate of occult HBV infection (HBV-DNA positive). However, the most significant finding of this study was that HBV-DNA was detected in 20.7% (11/53) patients in whom circulating anti-HBs antibodies were detectable. Of the HBV-DNA positives 55.6% (5 of 9) patients showed raised alanine aminotransferase levels while 66.7% (6 of 9) had a CD4^+ ^T cell counts below 200 cells/cu mm. The prevalence rates of HBV-DNA were comparable in both males (36.8%) and females (40%). The demographic and laboratory characteristics of HBV-DNA positive and negative patients were significantly indifferent (table 3).

## Discussion

Co-infection of HIV significantly modifies the natural history of HBV infection. Before availability of HAART it was associated with a higher chronicity rate of acute hepatitis B, higher levels of HBV replication, a lower rate of spontaneous loss of HBV antigens and a high rate of reactivation [2-4]. However, even after widespread institution of HAART, studies have shown increased number of deaths attributed to liver disease possibly due to prolonged survival and time for development of cirrhosis. Reports of flares of necro-inflammatory activity in liver are also documented due to restoration of adaptive HBV specific immune response and innate non-specific immune responses [4]. The treatment of HBV in HIV co-infected patients is complex because the drug(s) used are associated with drug-resistance, cross-resistance, hepatotoxicity and unresponsiveness. Another major concern is reactivation of HBV replication after withdrawal of dually active antiviral drugs leading to emergence of resistant strains.

Our study findings indicate that HIV-infected patients are at a higher risk of HBV co-infection in our set-up, as illustrated by the high prevalence of HBsAg (7.28%) in HIV positive patients in comparison to HIV negative patients (1.4%). Male patients were found more likely to be co-infected with HBV as compared to females (table. 1). This trend can be explained on the basis of higher rate of sexual promiscuity and other exposure risks in males. The majority of HIV-HBV co-infections were seen in the sexually active population indicating a common mode of transmission to these viruses, as has been previously documented [19]. Our study also shows a very high persistence of HBV genome (overall 37.5%) even in those patients who are HBsAg negative. The study further shows that like HBsAg other serological markers were also of lesser value to rule out the occult infection of HBV which is supported by the data that occult HBV infection was frequent in those who were positive for anti-HBc antibodies and also those who had significant levels of anti-HBs antibodies in their serum. Presence of Anti-HBc antibodies alone was also found associated with low CD4^+ ^cell counts (less than 200), even though these patients were treatment naive. Interestingly presence of naturally acquired antibodies to HBsAg (anti-HBs) did not exclude the existence of occult infection.

The clinical relevance of occult HBV infection in humans is not limited to blood banks but it is a still ongoing process. HBV particles may persist for decades after self-limited acute hepatitis and clinical recovery [20]. Thus, though occult infection alone may not have clinical consequences but may become injurious only when the virus is reactivated after immunosuppression [12,13]. A number of explanations for the persistence of HBV-DNA in HBsAg negative samples have been proposed, including the presence of HBV-DNA at a low copy number [21], genetic variations in the S gene [22] and the presence of immune complexes in which HBsAg may be hidden [23]. Many epidemiologic and molecular studies indicate that persistent HBV infection may have a critical role in the development of hepatocellular carcinoma in HBsAg-negative patients. Occult HBV infection and its potential oncogenicity are traditionally considered a consequence of the capacity of the virus to be integrated into the host genome, although many observations now show that free episomal HBV genomes may persist in the liver cells during occult infection. It may induce mild long lasting necroinflammation [20] and may progress to cirrhosis and hepatocellular carcinoma [24]. Studies have also shown that mutations in the hepatitis B X gene (HBx) protein modify the functioning of p53, a tumor suppressor gene [25]. It is still unclear if there is an altered prevalence of such mutations in occult HBV infections or if coinfection with HIV could play a catalytic role.

Though reactivation of HBV infection (in the absence of HBsAg) has been described anecdotally in HIV-infected patients following severe immune depletion and/or lamivudine withdrawal, yet the true prevalence and clinical impact of occult HBV infection in HIV- infected subjects is controversial due to lack of methodological standardisation and fewer prospective studies. The available data on prevalence of occult HBV are wildly divergent and inadequate. In fact, the published prevalence rates range from 0% to 89%, with a considerable number of other studies reporting results between these two extremes [26]. One basis for difference could be the difference in methodologies adopted by various workers and the number of samples tested. One recent study which tested a prospective cohort of 909 HIV positive patients for occult HBV showed a prevalence of 1.3% [17], but the sera were pooled in this study which makes it incomparable to ours. However, there is no study from Indian sub-continent to compare our results.

HBV-DNA detection has made significant impact on the management of these patients. According to treatment guidelines for HIV co-infected individuals, it is advocated that HBsAg positive patients should be treated in presence of elevated HBV-DNA, raised aminotransferases or significant hepatic fibrosis [27]. But, as yet no clear guidelines have been established for occult HBV co-infection in HIV positive patients. Moreover, the issue needs to be addressed whether to offer HBV vaccine to HBsAg negative HIV positive patients harbouring occult HBV infection. This study even though had some limitations, clearly indicates that routinely used serological markers of HBV infection do not rule out occult and ongoing Hepatitis B virus infection and emphasizes application of molecular methods for the detection of occult HBV infection in these patients. Some blood banks around the world have now routinely started nucleic acid testing (NAT) to ensure safe blood transfusion practices. However, this practice needs to be extended at every blood bank and specially when managing the HIV positive patients.

### Limitation of The study

A major limitation in our study was the small sample size and that it was a single time point testing without any follow-up. The sample size of the studied patients was small even though we have a large pool of patients due to cost constraints. The HBV-DNA estimation along with all serological marker costs us approximately US 100 per patients. We are in process of getting funds to test all our patients to assess the prevalence of occult HBV infection in all HIV positive patients. We also had limitation of not doing multivariate analysis of the data. Since it was a cross-sectional study we could not comment on the clinical significance of occult HBV in HIV patients included in our study. Lastly, since ours is a tertiary care referral centre, hence a patient selection bias cannot be completely ruled out.

## Conclusion

A sizeable number of HIV-HBV co-infected patients remain undiagnosed, if only conventional serological markers for HBV are used. The presence of anti-HBs antibodies is also an unreliable surrogate marker to rule out occult HBV infection. Though the most reliable method to diagnose occult HBV co-infection in HIV seropositive patients remains the detection of HBV DNA, it would not be practical yet to recommend this highly sensitive molecular assay for each and every patient in resource limited countries.

## Competing interests

The authors declare that they have no competing interests.

## Authors' contributions

SS was responsible for conceptualizing the study, arranging diagnostic services and facilities and critically reviewing the manuscript. SG analysed the data and prepared the draft of manuscript with data analysis and manuscript preparation. Both authors read and approved the manuscript.

## Pre-publication history

The pre-publication history for this paper can be accessed here:

http://www.biomedcentral.com/1471-2334/10/53/prepub

## References

[B1] Fauci AS Lane HC Braunwald E, Fauci AS, Kasper DL, Hauser SL, Longo DL, Jameson JL Human immunodeficiency virus (HIV) diseases: AIDS and related disorders Harrison's principles of internal medicine 2001 15 New York: McGraw-Hill 1852 1912

[B2] Bodsworth N Donovan B Nightingale BN The effects of concurrent human immunodeficiency virus infection on chronic hepatitis B: a study of 150 homosexual men J Infect Dis 1989 160 577 582 2571646 10.1093/infdis/160.4.577

[B3] Colin JF Cazals-Hatem D Loriot MA Martinot-Peignoux M Pham BN Auperin A Degott C Benhamou JP Erlinger S Valla D Marcellin P Influence of human immunodeficiency virus infection on chronic hepatitis B in homosexual men Hepatology 1999 29 1306 1310 10.1002/hep.510290447 10094979

[B4] Benhamou Y Antiretroviral therapy and HIV/hepatitis B virus coinfection Clin Infect Dis 2004 38 S98 S103 10.1086/381451 14986281

[B5] Haverkamp M Smit M Weersink A Boucher CA Hoepelman AIM The effect of lamivudine on the replication of hepatitis B virus in HIV infected patients depends on the host immune status (CD4+ cell count) AIDS 2003 17 1572 1574 10.1097/00002030-200307040-00023 12824802

[B6] Fang CT Chen PJ Chen MY Hung CC Chang SC Chang AL Chen DS Dynamics of plasma hepatitis B virus levels after highly active antiretroviral therapy in patients with HIV infection J Hepatol 2003 39 1028 1035 10.1016/S0168-8278(03)00416-1 14642622

[B7] Torbenson M Thomas DL Occult hepatitis B Lancet Infect Dis 2002 2 479 486 10.1016/S1473-3099(02)00345-6 12150847

[B8] Levicnik-Stezinar S Rahne-Potokar U Candotti D Lelie N Allain JP Anti-HBs positive occult hepatitis B virus carrier blood infectious in two transfusion recipients J Hepatol 2008 48 6 1022 5 10.1016/j.jhep.2008.02.016 18436328

[B9] Saraswat S Banerjee K Chaudhury N Mahant T Khandekar P Gupta RK Naik S Post-transfusion hepatitis type B following multiple transfusions of HBsAg-negative blood J Hepatol 1996 25 639 643 10.1016/S0168-8278(96)80232-7 8938539

[B10] Hoofnagle JH Seeff LD Bales ZB Zimmerman HJ Type B hepatitis after transfusion with blood containing antibody to hepatitis B core antigen N Engl J Med 1978 298 1379 1383 652005 10.1056/NEJM197806222982502

[B11] Chazouilleres O Mamish D Kim M Carey K Ferrell L Roberts JP Ascher NL Wright TL Occult hepatitis B virus as source of infection in liver transplant recipients Lancet 1994 343 142 146 10.1016/S0140-6736(94)90934-2 7904004

[B12] Grumayer ER Panzer S Ferenci P Gadner H Recurrence of hepatitis B in children with serologic evidence of past hepatitis B virus infection undergoing antileukemic chemiotherapy J Hepatol 1989 8 232 235 10.1016/0168-8278(89)90012-3 2715623

[B13] Waite J Gilson RJC Weller IVD Lacey CNJ Hambling MH Hawkins A Briggs M Tedder RS Hepatitis B virus reactivation or reinfection associated with HIV-1 infection AIDS 1988 2 443 448 10.1097/00002030-198812000-00006 3149492

[B14] Nunez M Rios P Perez-Olmeda M Soriano V Lack of 'occult' hepatitis B virus infection in HIV-infected patients AIDS 2002 16 2099 2101 10.1097/00002030-200210180-00024 12370518

[B15] Hofer M Joller-Jemelka H Grob P Frequent chronic hepatitis B virus infection in HIV-infected patients positive for antibody to hepatitis B core antigen only. Swiss Cohort Study Eur J Clin Microbiol Infect Dis 1998 17 6 13 10.1007/BF01584356 9512175

[B16] Piroth L Grappin M Buisson M Duong M Portier H Chavanet P Hepatitis B Virus seroconversion in HIV-HBV co-infected patients treated with highly active antiretroviral therapy J Acquir Immune Defic Syndr 2000 23 356 357 10836760 10.1097/00126334-200004010-00013

[B17] Shire NJ Rouster SD Stanford SD Blackard JT Martin CM Fichtenbaum CJ Sherman KE The prevalence and significance of occult hepatitis B virus in a prospective cohort of HIV-infected patients J Acquir Immune Defic Syndr 2007 44 3 309 14 10.1097/QAI.0b013e31802e29a9 17159656

[B18] Gupta S Singh S Hepatitis B and C virus co-infections in human immunodeficiency virus positive North Indian patients World J Gastroenterol 2006 12 42 6879 6883 17106941 10.3748/wjg.v12.i42.6879 PMC4087447

[B19] Gupta S Gupta R Joshi YK Singh S Role of Horizontal Transmission in Hepatitis B Virus spread among Household Contacts in North India Intervirology 2008 51 7 13 10.1159/000118790 18309243

[B20] Yotsuyanagi H Yasuda K Iino S Moriya K Shintani Y Fujie H Tsutsumi T Kimura S Koike K Persistent viremia after recovery from self-limited acute hepatitis B Hepatology 1998 27 1377 1382 10.1002/hep.510270526 9581694

[B21] Brechot C Thiers V Kremsdorf D Nalpas B Pol S Paterlini-Brechot P Persistent hepatitis B virus infection in subjects without hepatitis B surface antigen: clinically significant or purely "occult"? Hepatology 2001 34 194 203 10.1053/jhep.2001.25172 11431751

[B22] Carman WF The clinical significance of surface antigen variants of hepatitis B virus J Viral Hepat 1997 4 Suppl 1 11 20 10.1111/j.1365-2893.1997.tb00155.x 9097273

[B23] Liang TJ Blum HE Wands JR Characterization and biological properties of a hepatitis B virus isolated from a patient without hepatitis B virus serologic markers Hepatology 1990 12 204 212 10.1002/hep.1840120205 2167867

[B24] Squadrito G Pollicino T Cacciola I Caccamo G Villari D La Masa T Restuccia T Cucinotta E Scisca C Magazzu D Raimondo G Occult hepatitis B virus infection is associated with the development of hepatocellular carcinoma in chronic hepatitis C patients Cancer 2006 106 1326 1330 10.1002/cncr.21702 16453330

[B25] Huo TI Wang XW Forgues M Wu CG Spillare EA Giannini C Brechot C Harris CC Hepatitis B virus X mutants derived from human hepatocellular carcinoma retain the ability to abrogate p53-induced apoptosis Oncogene 2001 20 3620 3628 10.1038/sj.onc.1204495 11439325

[B26] Raimondo G Pollicino T Cacciola I Squadrito G Occult hepatitis B virus infection J Hepatol 2007 46 1 160 70 10.1016/j.jhep.2006.10.007 17112622

[B27] Soriano V Puoti M Peters M Benhamou Y Sulkowski M Zoulim F Mauss S Rockstroh J Care of HIV patients with chronic hepatitis B: updated Recommendations from the HIV-Hepatitis B Virus International Panel AIDS 2008 22 1399 1410 10.1097/QAD.0b013e3282f8b46f 18614862

